# Developing High-Capacity Solid “Molecular Basket” Sorbents for Selective CO_2_ Capture and Separation

**DOI:** 10.1021/acs.accounts.3c00444

**Published:** 2023-11-20

**Authors:** Xiaoxing Wang, Chunshan Song

**Affiliations:** † EMS Energy Institute, Departments of Energy and Mineral Engineering and of Chemical Engineering, The Pennsylvania State University, University Park, Pennsylvania 16802, United States; ‡ Department of Chemistry, Faculty of Science, The Chinese University of Hong Kong, Shatin, NT, Hong Kong 999077, China

## Abstract

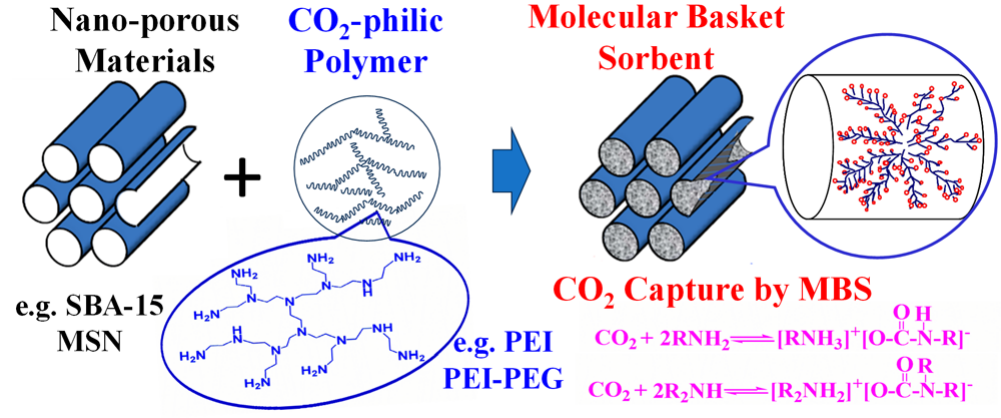

Since carbon-based energy continues to dominate (over 80%) the global primary energy supply, carbon dioxide capture, utilization, and sequestration (CCUS) is deemed to be a promising and viable option to mitigate greenhouse gas emissions and climate change, for which CO_2_ capture is critical to the overall success of CCUS. Although liquid amine scrubbing is a mature technology for carbon capture, it is energy-intensive and costly due to energy consumption in solvent heating and water evaporation apart from the energy needed to break amine–CO_2_ bonding. To address this challenge, Song’s group developed a new design approach for adsorptive CO_2_ capture and separation, namely, “molecular basket” sorbents (MBS), without the need for dealing with solvent heating and water evaporation. The solid MBS consisting of polymeric amines (such as PEI) immobilized into nanoporous materials (such as SBA-15) possesses a high capacity for CO_2_ capture with high selectivity, fast kinetics, and good regenerability. Consequently, MBS can greatly reduce energy consumption and carbon capture cost. Conventional adsorbents such as zeolites, activated carbon, alumina, and silica have low adsorption capacities, and their use of CO_2_ adsorption requires prior removal of moisture and cooling of flue gas (∼35 °C). On the contrast, the CO_2_ sorption capacity of MBS can even be promoted by the presence of moisture/steam and reaches the best performance closer to flue gas temperature (∼75 °C). This Account presents an overview of our research progress in the material development and fundamental understanding of MBS for CO_2_ capture and the separation of CO_2_ from various gas streams. It begins with an illustration of the MBS concept, followed by efforts to improve the performance and pilot-scale demonstration of MBS for CO_2_ capture. With the systematic characterization of MBS by various ex situ and in situ techniques, a better understanding is developed for the CO_2_ sorption process mechanistically. Furthermore, this Account demonstrates how the fundamental understanding of the CO_2_ sorption mechanism promotes the further development of more robust and advanced sorbent materials with improved CO_2_ sorption capacity, kinetics of sorption and desorption, and cyclic stability. Finally, an outlook is provided for the future design and development of novel sorbent materials and the CO_2_ sorption process for various gas streams including flue gas, biogas, air, and hydrogen streams.

## Key References

- XuX.; SongC.; AndresenJ. M.; MillerB. G.; ScaroniA. W.Novel Polyethylenimine-Modified Mesoporous Molecular Sieve of MCM-41 Type as High-Capacity Adsorbent for CO_2_ Capture. Energy Fuels2002, 16, 1463–1469. 10.1021/ef020058u(1)*This is the first peer-reviewed publication on molecular basket sorbents including materials preparation, characterization, and their excellent performance for CO*_2_*adsorption from flue gas, though the concept was first proposed in a conference proceeding in 2001*.
- MaX.; WangX.; SongC.“Molecular Basket” Sorbents for Separation of CO_2_ and H_2_S from Various Gas Streams. J. Am. Chem. Soc.2009, 131, 5777–578310.1021/ja807410519348482 .^2^*Here, we developed a new generation of molecular basket sorbents with much improved CO*_*2*_*capture capacity and identified its unique temperature dependence for CO*_*2*_*capture and its expanded application for the adsorptive separation of CO*_*2*_*and H*_*2*_*S*.
- WangX.; SongC.Temperature-programmed desorption of CO_2_ from polyethylenimine-loaded SBA-15 as molecular basket sorbents. Catal. Today2012, 194, 44–5210.1016/j.cattod.2012.08.008.^3^*In this work, we used the temperature-programmed desorption technique to study the CO*_*2*_*sorption mechanism over the molecular basket sorbents and proposed a two-layer model for the first time to explain the CO*_*2*_*sorption process*.
- LouF.; ZhangA.; ZhangG.; RenL.; GuoX.; SongC.Enhanced kinetics for CO_2_ sorption in amine-functionalized mesoporous silica nanosphere with inverted cone-shaped pore structure. Appl. Energy2020, 264, 11463710.1016/j.apenergy.2020.114637.^4^*In this work, we show that a novel type of pore structure, inverted cone-shaped pore structure for nanoporous silica, can impart a much faster sorption rate and a significantly higher desorption rate for PEI-based molecular basket sorbents for CO*_*2*_*capture and separation*.

## Introduction

Carbon dioxide is a typical greenhouse gas which can trap heat to sustain life on earth but can also cause global warming. The atmospheric CO_2_ level has risen rapidly to over 415 ppm due to increasing global carbon emissions, which reached 34.9 Gt-CO_2_ in 2021.^5,6^ Thus, it is of great importance and urgency to mitigate CO_2_ emissions. Among various developing technologies, CO_2_ capture, utilization, and sequestration (CCUS) is considered to be a viable option to significantly mitigate CO_2_ emissions while allowing the energy transition to take place in the foreseeable future toward carbon neutrality.

Amine scrubbing has been used to capture acid gases from natural gas and hydrogen in industry since 1930.^7^ However, its application to the capture of CO_2_ from flue gas is very energy-intensive.^7^ Flue gas contains low partial pressure CO_2_ (4–15%) at a relatively high temperature. About 3.6–4.0 GJ is consumed per ton of CO_2_ captured with 30% MEA solution; roughly 15% of the energy is consumed in heating the aqueous solution, 35% consumed in water evaporation, and 50% is consumed in breaking up the primary amine–CO_2_ bonding at an elevated temperature such as 120 °C. The loss of amine from degradation and solvent evaporation and the corrosive nature of liquid amine also present processing problems. Thus, developing a more energy-efficient and cost-effective technology for CO_2_ capture is highly desired and still a major global challenge.

**Figure 1:**
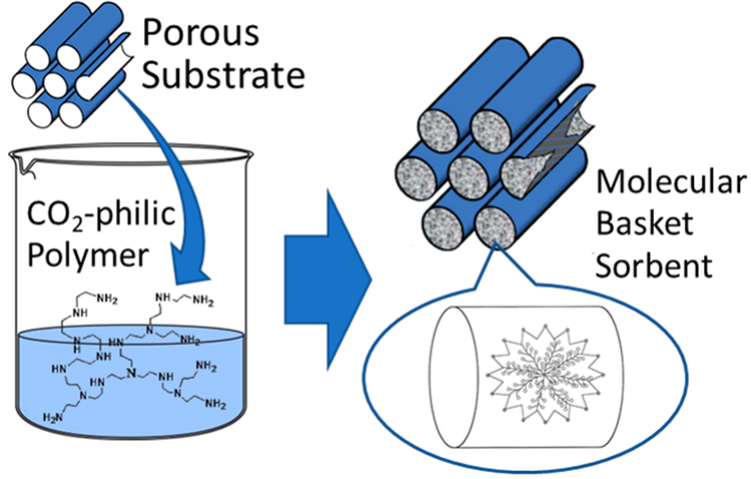
Concept and preparation scheme of the molecular basket sorbent for CO_2_ capture.

Our initial question toward dramatically reducing energy consumption was, can we eliminate solvent heating and remove water evaporation (eliminate up to 50% of energy consumption in liquid amine scrubbing) and further reduce the binding strength between amine and CO_2_? The question led us to propose a new concept called the “molecular basket” sorbent (MBS) as a novel type of solid material for CO_2_ capture with high capacity and high selectivity in 2001,^8^ and we reported its excellent performance in 2002.^1^ In this concept, a nanoporous material with a large pore volume and surface area such as MCM-41 or SBA-15 serves as the “basket” and a CO_2_-philic polymer containing more secondary amine encapsulated within pore channels serves as the “molecular leaf” providing numerous sites to capture CO_2_ (Figure 1), which is distinctly different from conventional adsorbents such as activated carbon, alumina, and silica.

The molecular basket sorbent (MBS) possesses the merits of both a nanoporous support and an amine polymer. On one hand, using an amine polymer can largely increase the accessible sorption sites per weight/volume of sorbent. On the other hand, using a high-surface-area material with a large pore volume increases the interface between CO_2_ and amine polymer, thus improving the mass transfer. As a result, high capacity, fast kinetics, and high selectivity may be achieved. It eliminates the parasitic energy loss induced by water solvent, which accounts for around 40% of the total energy consumption in liquid amine scrubbing.^7^

Since then, amine-based solid sorbents have received increasing attention worldwide. The related research and publications increased considerably, as they demonstrate many advantages including high CO_2_ capacity and selectivity, fast kinetics, good stability, and tolerance to water with no or less corrosion of the equipment.^2,9−12^ They can also be applied to other applications such as biogas upgrading^13−15^ and hydrogen purification.^16−20^ This Account summarizes our efforts in developing MBSs for CO_2_ capture and separation from various gas streams, from fundamental work including qualitative and quantitative characterization and sorption mechanism clarification^1−3,10,12,21−23^ to applications including CO_2_ capture from real flue gases and bench-scale pilot testing.^4,24−38^

## Fundamental Understanding of MBS and Its CO_2_ Sorption

Normally, the adsorption capacity decreases with temperature as adsorption is an exothermic process. However, over the MBS with PEI loading of 50 wt % or higher, we observed that the CO_2_ capacity increases with increasing temperature and reaches its maximum at a given temperature and then decreases with further increasing temperature. It was first reported that over PEI-50/MCM-41, the CO_2_ capacity was 44, 112, and 110 mg of CO_2_/g of sorbent at 50, 75, and 100 °C, respectively.^1,39^ In the following studies, we observed a similar temperature dependence on various MBSs synthesized with different supports including SBA-15,^2,3,22,23^ TUD-1,^32^ silica gel,^40^ various three-dimensional mesoporous materials,^33^ fumed silica,^35^ clay material,^32^ and carbon materials.^27,28^ Such a temperature dependence has also been observed by many others.^1,21,39,41,42^ All of these reflect that the temperature dependence is a universal fact and reveal that MBSs behave differently from conventional adsorbents, which merits further study to improve our understanding of MBSs and the associated CO_2_ sorption process.

We proposed the hypothesis that the sorption of CO_2_ over MBSs is controlled by both thermodynamics and kinetics. At low temperature, it is thermodynamically favorable. However, due to the kinetic limitation (i.e., diffusion barrier), not all sorption sites are available for CO_2_. At elevated temperature, such as 75 °C, the kinetic limitation can be largely overcome, resulting in an increase in CO_2_ sorption, though the adsorption itself is less thermodynamically favorable. When the temperature is too high (e.g., 100 °C), however, CO_2_ sorption becomes thermodynamically unfavorable and thus the capacity decreases.

**Figure 2:**
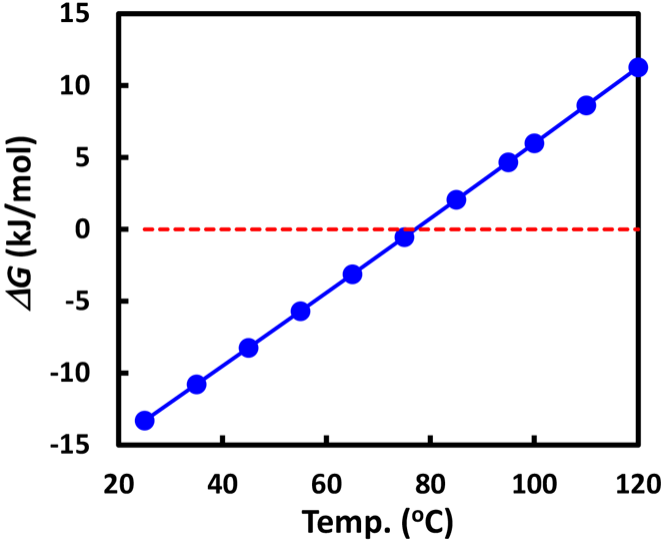
Estimated Gibbs free energy as a function of temperature for CO_2_ sorption over MBS such as PEI/SBA-15.

We first verify that CO_2_ sorption on MBS is thermodynamically viable at an elevated temperature such as 75 °C, which is determined by the change in the Gibbs free energy (*ΔG*). For CO_2_ sorption, *ΔG* can be calculated with the following equation
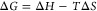
1where *ΔH* (kJ/mol) and *ΔS* (J/K/mol) are the changes in enthalpy and entropy during the process, respectively, and *T* is the sorption temperature (K). *ΔH* is the adsorption heat, which is ca. −77 kJ/mol on average for CO_2_ sorption over supported amine adsorbents (typically in the range of 50–105 kJ/mol^43^). The entropy (*S*) of CO_2_ gas at different temperatures can be estimated with eq 2(44)
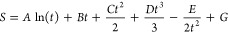
2
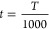
3where *A*, *B*, *C*, *D*, *E*, and *G* are constants whose values can be found in the literature.^44^ After adsorption, the entropy of the CO_2_ molecules becomes zero. Consequently, we can estimate the *ΔG* for CO_2_ sorption at different temperatures (Figure 2). *ΔG* increases with temperature and reaches zero at a temperature of ca. 80 °C, indicating that CO_2_ sorption is thermodynamically favorable below 80 °C.

**Figure 3:**
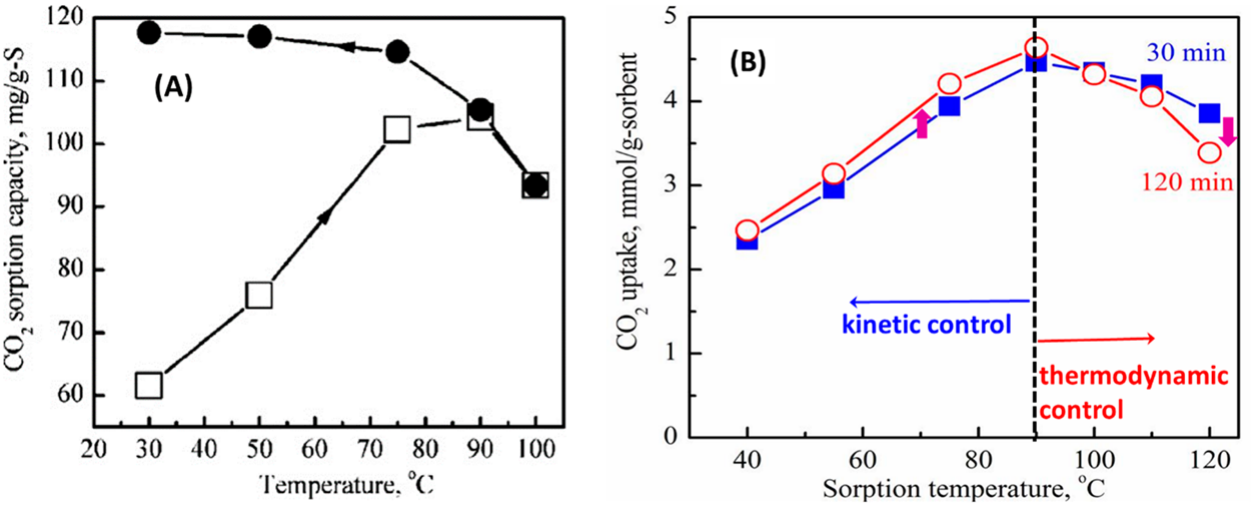
CO_2_ capacity of PEI-50/SBA-15 as a function of (A) temperature with temperature increase (□) and decrease (●) [reproduced with permission from ref (23), copyright 2013 Elsevier] and (B) at 75 °C with different sorption times of (■) 30 min and (○) 120 min [reproduced with permission from ref (3), copyright 2012 Elsevier].

It is known that temperature can influence not only the thermodynamics of the process but also the kinetics. In Figure 3A, the CO_2_ capacity was measured step by step with increasing temperature from 30 to 100 °C and then with temperature decreasing. We can see an increasing trend in CO_2_ capacity with temperature, reaching the optimum at 75–90 °C and then decreasing again at 100 °C. However, when the temperature decreased from 100 °C stepwise down to 30 °C, the capacity showed an increasing trend with the decrease in temperature, giving the highest CO_2_ capacity at 30 °C. This suggests that low capacity at low temperature is caused by the lack of access to the amino functional sites on the polymeric PEI chains causing slow kinetics, which is commonly referred to as the diffusion barrier.

We then investigated the influence of the sorption time. As shown in Figure 3B, at temperature below 100 °C, the CO_2_ capacity increases with increasing sorption time, indicating the longer time required to reach CO_2_ sorption equilibrium. In contrast, at temperatures over 100 °C, the capacity drops with extended sorption time, reaching the desorption equilibrium. Both experiments in Figure 3 clearly indicate that kinetics plays a crucial role in determining the CO_2_ capacity of MBS with temperature.

Why does it take a longer time to reach equilibrium for CO_2_ sorption on MBS at low temperature? Of course, low temperature is one reason. But more intrinsically, it can be attributed to the MBS structure, particularly PEI inside the support’s pore channels. Thus, we applied various techniques including basic methods such as N_2_ physisorption and XRD and some advanced methods such as small-angle X-ray scattering (SAXS),^23^ FTIR,^10,22^ temperature-programmed desorption (TPD),^3^ STEM, and small-angle neutron scattering (SANS)^45^ to characterize MBS. XRD confirms the perseverance of the support structure. N_2_ physisorption shows that the surface area and pore volume decrease with PEI loading, whereas the pore diameter does not change much, until the loading amount is higher than 50 wt %.^23,32^ This indicates that at high PEI loading the loaded PEI may fill the inside of the pore channels rather than coating the pore walls.

**Figure 4:**
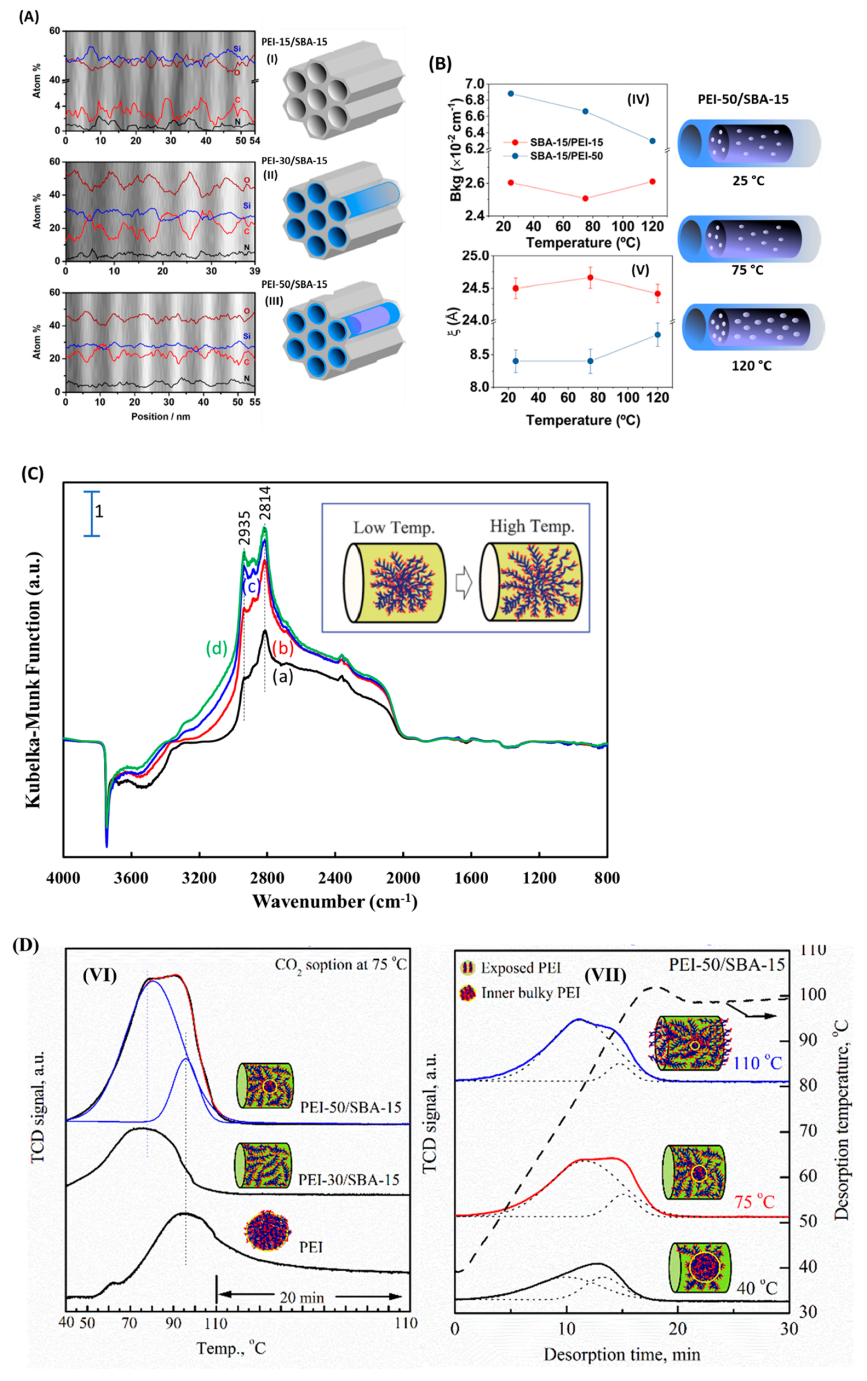
(A) STEM-EDS images and atomic percentages of Si, O, C, and N elements for samples (I) PEI-15/SBAA-15, (II) PEI-30/SBA-15, and (III) PEI-50/SBA-15. (B) SANS results of the (IV) scattering background, and (V) scattering correlation length as a function of temperature for the PEI-50/SBA-15 sample in vacuum at 25, 57, and 120 °C. (White dots represent the voids generated due to PEI loading and swelling.) [Reproduced with permission from ref (45), copyright 2019 American Chemical Society.] (C) FTIR spectra of PEI-50/SBA-15 at (a) 30 °C (black), (b) 50 °C (red), (c) 75 °C (blue), and (d) 100 °C (olive) under UHP N_2_ flow with SBA-15 as the background (i.e., subtracting the SBA-15 spectrum) (reproduced with permission from ref (22), copyright 2012 Royal Society of Chemistry). (D) CO_2_-TPD profiles of (VI) PEI, PEI-30/SBA-15, and PEI-50/SBA-15 at 75 °C and (VII) PEI-50/SBA-15 at sorption temperatures of 40, 75, and 110 °C [reproduced with permission from ref (12), copyright 2019 Springer].

STEM-EDS shows the distribution of Si, O, C, and N elements of the PEI-15/SBA-15, PEI-30/SBA-15, and PEI-50/SBA-15 sorbents across the pore channels (Figure 4A).^45^ Here, the bright and dark areas reflect the silica skeleton and mesopore channels of the SBA-15 support, respectively. The presence of C and N elements is attributed to PEI polymer, and Si and O elements represent the SBA-15 support. PEI-15/SBA-15 has a relatively lower carbon percentage than PEI-30/SBA-15, which is reasonable because of its lower PEI content. The carbon distribution of both samples is less homogeneous than that of PEI-50/SBA-15 along the radial direction. A sharp increase or decrease in the carbon percentage occurs at the interface between the silica skeleton (bright area) and mesopores (dark area). It indicates that PEI is initially dispersed on the walls of mesopores and then begins to fill into and plug the mesopore space with the increase in PEI loading (see Figure 4A for a pore-filling illustration). Such an observation is consistent with the recent discovery of the PEI distribution within SBA-15 using the SANS technique reported by Holewinski et al.^46,47^ The aggregated PEI plugging pore channels could be the origin of the diffusion barrier for the CO_2_ sorption.

An in situ SANS technique was applied to investigate how temperature affects PEI swelling/shrinking inside the pores. Neutrons are sensitive to the hydrogen element; thus, the background signal of SANS can be used as an indicator of PEI swelling. As shown in Figure 4B, the scattering background decreases with an increase in temperature for PEI-50/SBA-15 (blue dots in Figure 4B–IV), indicating PEI swelling with temperature. In contrast, the change is negligible for PEI-15/SBA-15 (red dots in Figure 4B–IV). Combined with results from Figure 4A, it suggests that PEI functioning as plugs inside the mesopores is swelling because of the temperature. Furthermore, the scattering correlation length ξ which represents the average size of disordered intramolecular voids between PEI chains can also be used to indicate the change in the PEI status. As shown in Figure 4B–V, the ξ at 75 °C is almost the same as that at 25 °C but increases from ∼8.4 to ∼8.8 Å when the temperature increases from 75 to 120 °C. The increase in the intramolecular voids reflects the space increase between PEI molecules. In other words, it provides evidence of the swelling of PEI at elevated temperature.^45^

The swelling of PEI was also confirmed by FTIR. As shown in Figure 4C, the intensities of the IR bands at 2935 and 2814 cm^–1^ corresponding to the asymmetric and symmetric stretching of PEI’s CH_2_–CH_2_ chain increase with temperature, suggesting that the vibration of the PEI CH_2_–CH_2_ chain becomes stronger. In other words, PEI is swelling with the temperature, as illustrated in the inset of Figure 4C. It is consistent with the SANS observation (Figure 4B). The swelling makes more of the intramolecular space accessible, leading to more amine sites open for CO_2_ sorption and thus an increase in capacity.

We used the TPD technique to measure the change in the CO_2_ sorption capacity with temperature. The CO_2_-TPD profile of PEI-50/SBA-15 can be deconvoluted into two peaks (i.e., a low-temperature desorption peak, LTDP, and a high-temperature desorption peak, HTDP), corresponding to the loosened and aggregated parts of PEI, respectively (Figure 4D). PEI-30/SBA-15 shows only one LTDP corresponding to the loosened PEI, whereas the aggregated bulk PEI gives HTDP. Over PEI-50/SBA-15, with the adsorption temperature increasing from 40 to 110 °C, the LTDP increases while the HTDP decreases. This suggests that with increasing temperature, the loosened part of PEI increases while the aggregated bulk PEI part decreases. Since the overall amount of PEI does not change with temperature, the results reflect that PEI swells with temperature, alleviating the diffusion barrier and exposing more accessible sites for CO_2_ sorption. This explains the unique temperature dependence of CO_2_ sorption over MBSs and supports our hypothesis of the CO_2_ sorption mechanism.

## Development and Application of MBSs

### Sorbent Development Approaches

As mentioned above, MBS consists of a nanoporous substrate and a CO_2_-philic polymer. Both components play a crucial role in determining the performance of MBSs for CO_2_ sorption including capacity, selectivity, kinetics, stability, regenerability, and the overall cost of the CO_2_ capture process. Consequently, our development of MBSs has been focused on both support materials and polymer compounds.

We first used mesoporous MCM-41 as the support and successfully obtained a high CO_2_ capacity (90 mg of CO_2_/g of sorbent) and a selectivity (CO_2_/N_2_ > 1000)^1,21^ which is much higher than those of most conventional adsorbents such as activated carbons and zeolites. Compared to MCM-41, SBA-15 has a larger surface area, pore volume, and pore size, which would facilitate the PEI dispersion and the CO_2_ diffusion. With SBA-15, we did obtain a better CO_2_ capacity, 140 mg of CO_2_/g of sorbent under the same condition.^2^ Moreover, the CO_2_ sorption rate of PEI-50/SBA-15 is 1.97 mg/s/g of PEI, 18% higher than that of PEI-50/MCM-41 (1.67 mg/s/g of PEI).^23^

**Figure 5:**
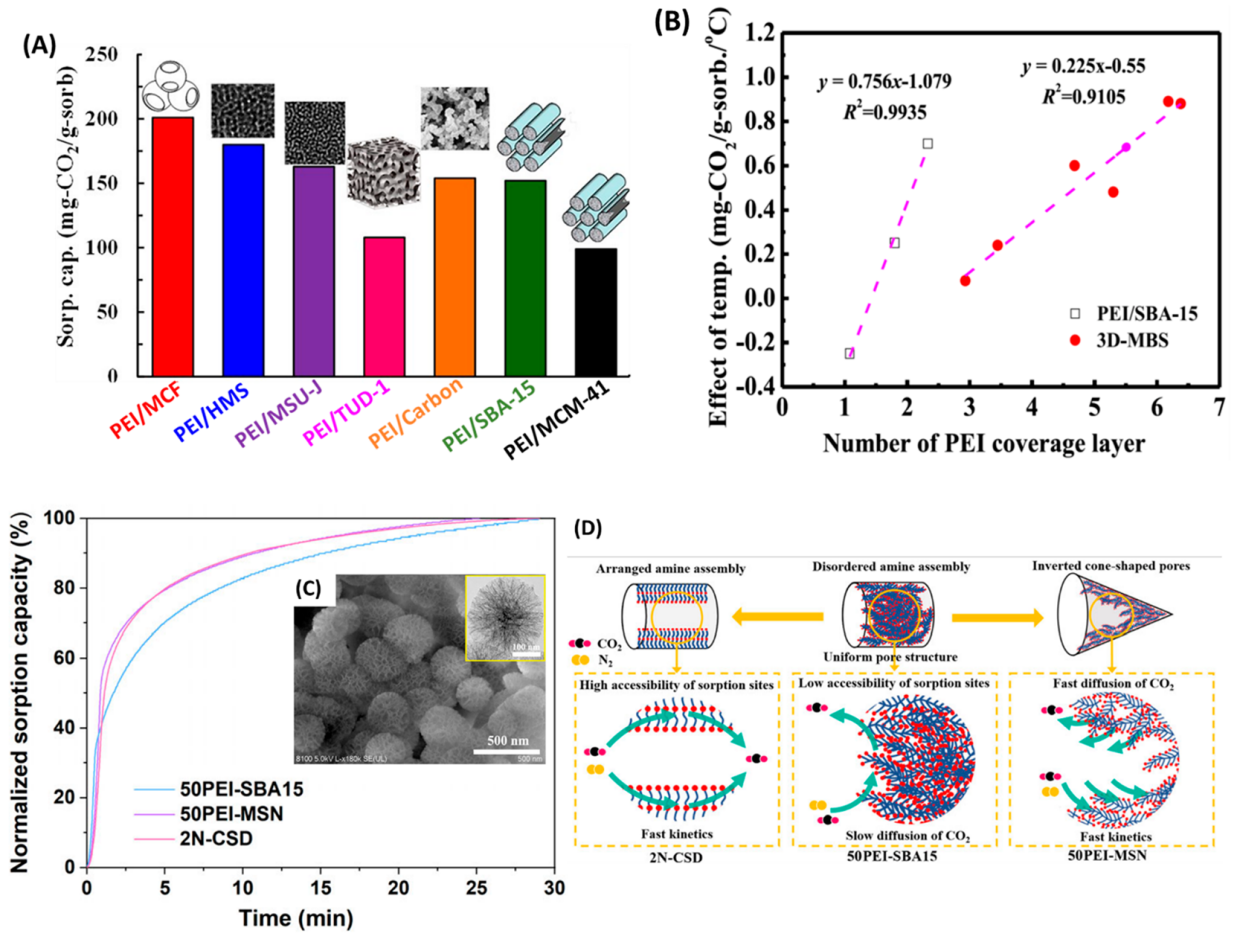
(A) Comparison of CO_2_ sorption capacity of 3-D MBS versus PEI(65)/CB, PEI(65)/SBA-15, and PEI(60)/MCM-41 [reproduced with permission from ref (31), copyright 2014 Elsevier]. (B) Effect of temperature on CO_2_ sorption capacity versus PEI loading on 3-D MBS and PEI/SBA-15 [reproduced with permission from ref (35), copyright 2017 Elsevier]. (C) Normalized sorption capacity of 2N-CSD, PEI-50/MSN, and PEI-50/SBA-15 at 30 °C (insets: TEM and SEM images of PEI-50/MSN). (D) Schematic illustration of CO_2_ sorption over 2N-CSD, PEI-50/MSN, and PEI-50/SBA-15 sorbents [reproduced with permission from ref (38), copyright 2021 Springer].

According to our earlier discussion, reducing the diffusion barrier is crucial to MBS performance. A support with a three-dimensional (3-D) pore structure could promote CO_2_ diffusion from all directions compared to those with a one-dimensional (1-D) pore structure. Thus, we prepared a series of MBSs based on 3-D mesoporous materials including MCF, HMS, MSU-J, and TUD-1 for CO_2_ sorption.^32,33^ The PEI dispersion was better with the 3-D pore structure, leading to less restriction for CO_2_ diffusion and more exposed amine sites for CO_2_ sorption. Consequently, the 3-D MBSs exhibited a significantly higher CO_2_ sorption capacity and a faster sorption rate compared with those of 1-D MBSs (Figure 5A). Additionally, the temperature dependence on 3-D MBS is much less than that of 1-D MBS, which can also be attributed to the 3-D pore structure facilitating CO_2_ diffusion and promoting PEI dispersion (Figure 5B; the definition and details of the number of PEI coverage layers can found in ref (38)).

The shape of the pore can also influence the MBS performance. In a recent work, we developed MBS with inverted-cone-shaped pores in a mesoporous silica nanosphere (MSN) loaded with PEI (PEI/MSN)^4^ and with an arranged amine assembly at nanoscale (2N-CSD) structure for CO_2_ capture.^38^ The PEI/MSN sorbents showed significantly faster kinetics of sorption and desorption along with improved capacity compared with PEI-50/SBA-15 at all temperatures studied. Even at 30 °C, PEI/MSN and 2N-CSD showed higher CO_2_ capacity and faster average CO_2_ sorption rates (Figure 5C). Higher amine efficiency was obtained over 2N-CSD, showing a much smaller diffusion barrier from PEI as illustrated in Figure 5D. This is an example showing the success of sorbent development based on a fundamental understanding of the CO_2_ sorption process.

One disadvantage of these nanoporous materials is their high cost and commercial unavailability.^40^ Thus, we have also explored inexpensive materials such as silica gel (SG),^40^ fumed silica (FS),^33,35,36^ bentonite,^29^ zeolite,^20^ and carbons^27,28^ for preparing MBS. The selected sorbents not only can exhibit a comparable weight-based capacity but also could possess a higher volume-based capacity. For example, the volumetric capacity of PEI-50/SG is 83 mg of CO_2_/cm^3^ of sorbent, which is about 2.6 times higher than that of PEI-50/SBA-15.^40^ With higher volumetric capacity, the overall size of the CO_2_ capture processor can be more compact, saving space and equipment.

The amine polymer itself is another critical aspect. We examined the effect of PEI properties including PEI type, molecular weight (MW), structure, and nitrogen density on the CO_2_ sorption performance.^34,48^ Linear PEI has some advantages over branched PEI, including higher CO_2_ capacity,^48^ faster kinetics and a lower heat of adsorption/desorption,^49^ and better cyclic stability.^49,50^ However, due to its extremely low availability and high cost, the application of linear PEI is significantly limited. PEI with low MW works better than that with high MW.^34,48^ This can be explained by the difference in the amine structure. The basicity of the amine group decreases as primary amine > secondary amine > tertiary amine, as does its ability to react with CO_2_. Linear and low-MW PEI contain relatively more secondary and primary amine groups than branched and high-MW PEI, thus exhibiting better performance for CO_2_ sorption. Another reason is the viscosity. PEI with high MW tends to have higher viscosity and thus a higher barrier for CO_2_ diffusion, leading to lower capacity and slower sorption kinetics.

**Figure 6:**
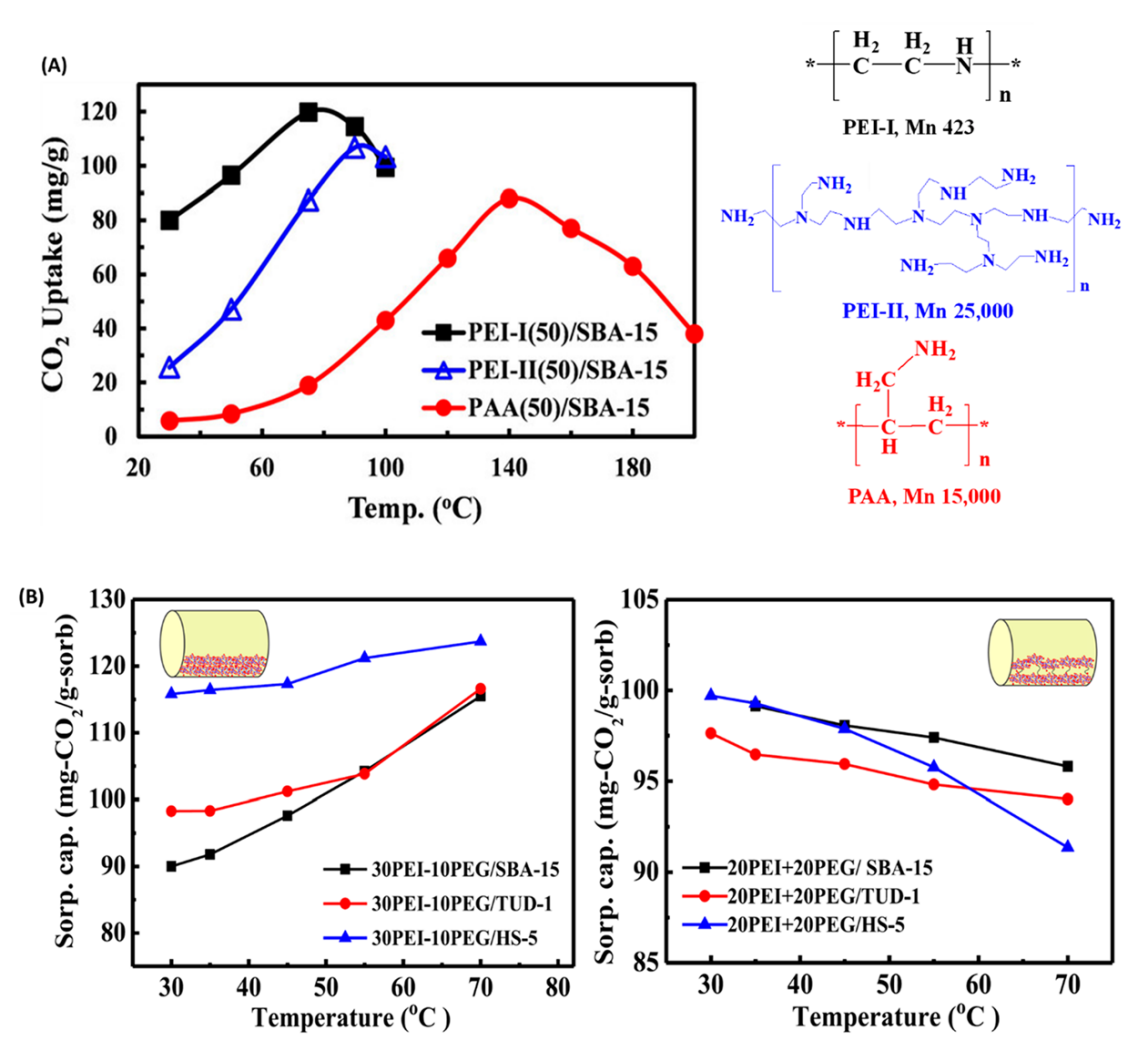
(A) CO_2_ capacity as a function of sorption temperature over PEI-I(50)/SBA-15, PEI-II(50)/SBA-15, and PAA(50)/SBA-15 sorbents [reproduced with permission from ref (34), copyright 2017 Wiley]. (B) CO_2_ sorption capacity of samples with (left) 10 wt % and (right) 20 wt % PEG addition over three MBS at different temperatures [reproduced with permission from ref (35), copyright 2017 Elsevier].

We examined another amine polymer, polyallylamine (PAA), which has a linear structure with primary amine groups on the branch only (Figure 6A).^34^ The capacity of PAA-MBS is generally lower than that of PEI-MBS, which is likely due to its relatively lower amine density on a weight basis. All of the sorbents exhibited the trend of increasing CO_2_ capacity with an increase in temperature, but their optimum temperatures are different. They are 75, 90, and 140 °C for PEI-I(50)/SBA-15, PEI-II(50)/SBA-15, and PAA(50)/SBA-15, respectively. This could be ascribed to the increased diffusion barrier due to the increased hydrogen bonding and rigid structure from linear low-MW PEI to high-MW branched PEI and further to PAA.^34^

Alleviating PEI aggregation inside pores can increase the number of accessible amine sites and promote the mass transfer rate, thus promoting the CO_2_ capacity, sorption kinetics, and amine utilization efficiency. We first developed a strategy to incorporate a structural modifier which could reduce PEI aggregation by introducing an additive, e.g., poly(ethylene glycol) (PEG), to PEI.^21^ With the addition of PEG, the CO_2_ capacity of PEI/MCM-41 increased from 68.7 to 77.1 mg of CO_2_/g of sorbent. Later, we systematically studied the effect of PEG addition on the CO_2_ capacity of PEI/SBA-15, PEI/TUD-1, and PEI/HS-5 sorbents.^35^ The promotion effect of PEG was further confirmed. At 10 wt % PEG addition, increasing temperature still exhibits a positive effect on CO_2_ capacity (Figure 6B). However, when 20 wt % PEG is added, it exhibits a negative impact; i.e., the CO_2_ capacity decreases with temperature. This suggests that the addition of PEG loosens the PEI aggregation and makes PEI layers more separated, resulting in a significant reduction in the diffusion barrier. Consequently, temperature displays a negative influence on the sorption capacity. Jones et al.^51^ reported the same PEG promotion effect through PEI–PEG intermolecular interaction over the supported PEI sorbents.

Beside PEG, other additives have also been investigated. To alter the amine–CO_2_ reaction chemistry, we added a small amount of potassium carbonate to PEI/FS.^33^ Both the gravimetric and volumetric capacities increased greatly with the addition of 4–6 wt % potassium carbonate. Furthermore, the addition of K_2_CO_3_ improved the amine efficiency and cyclic stability. Choi et al. first used PEI and a 3-aminopropyltriethoxysilane (APTES) mixture impregnated silica sorbent for CO_2_ capture from air, showing increased sorption kinetics and improved thermal/cyclic stability in comparison with the sorbent without APTES, although their CO_2_ capacity was similar.^52^ In contrast, we introduced APTES to modify the surface of the FS support, which appears to generate more intramolecular spaces within PEI/FS.^36^ The addition of APTES increased the capacity by 44% and the amine efficiency by over 25%. Both the sorption and desorption rates were promoted 1.23- and 1.61-fold as well. Choi et al.^53,54^ reported 1,2-epoxybutane (EB) to be an effective additive to significantly improve the long-term stability and oxidation resistance of supported PEI sorbents, although it decreases the total CO_2_ capacity of the sorbent.

### Exploration of Practical Application

Considering the urgency of the environmental impact and importance of CO_2_ capture from various gas streams at large, evaluating the sorbent performance with real flue gas is crucial for practical application and commercialization. Thus, we examined the first generation of MBS, PEI/MCM-41, for CO_2_ capture from a pilot-scale plant natural gas-fired boiler flue gas containing 7.4–7.7% CO_2_, 14.6% H_2_O, ∼4.45% O_2_, 200–300 ppm of CO, 60–70 ppm of NO_*x*_, and 73–74% N_2_.^24^ The study showed that CO_2_ can be selectively captured from real flue gas with fast sorption/desorption kinetics. The presence of moisture promoted the CO_2_ sorption. The sorbent was largely regenerable and stable. Although NO_*x*_ was also adsorbed, the amount of adsorbed CO_2_ was about 3,000 times larger. It was also found that very little NO_*x*_ was desorbed in regeneration, indicating that the presence of NO_*x*_ may degrade MBS in the long term.

**Figure 7:**
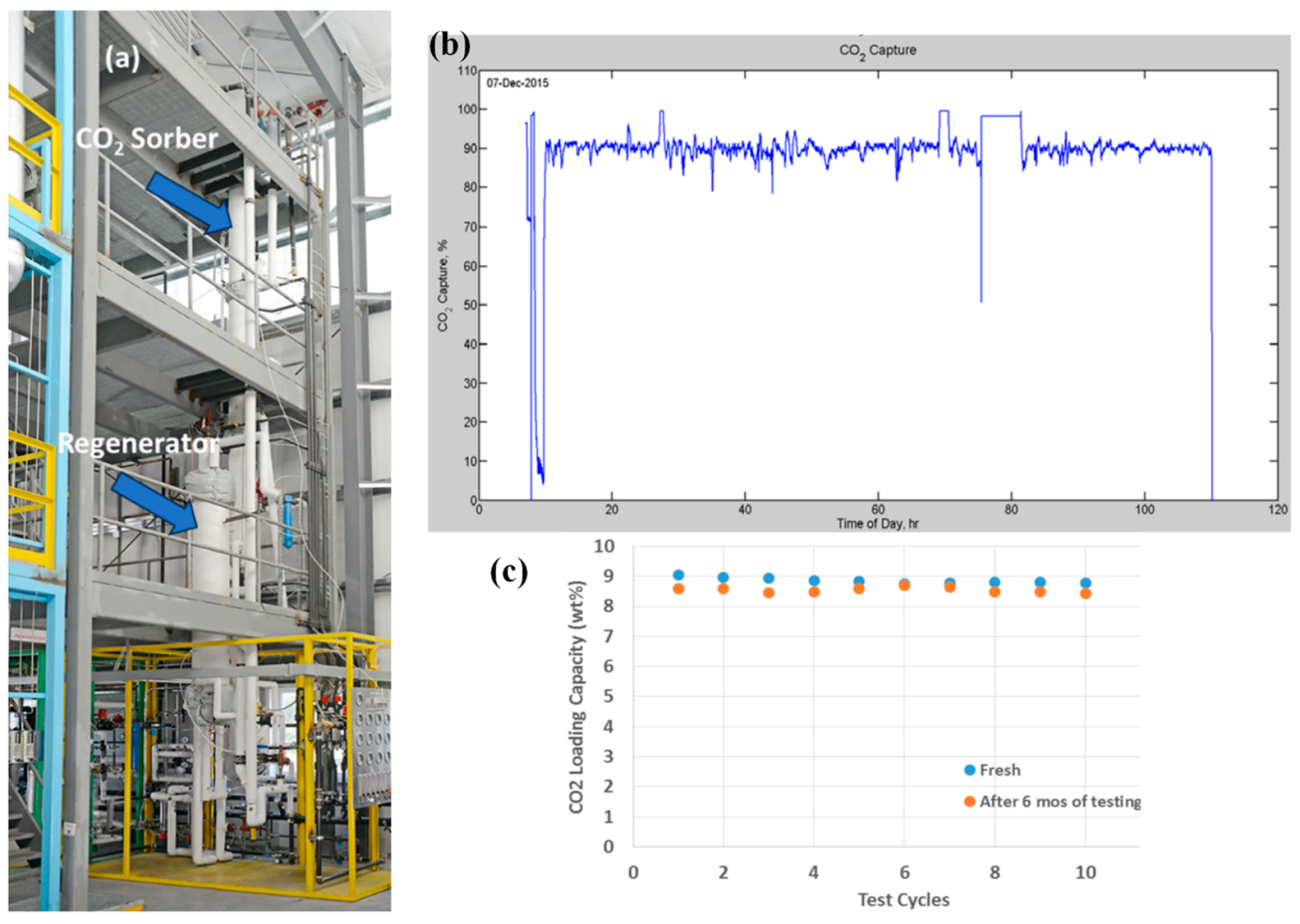
(a) Photo of bench-scale contract evaluation system containing two fluidized, moving-bed reactors (i.e., sorber and regenerator). (b) Measured rate of CO_2_ capture during the 100 h test in the bench-scale system. (c) MBS sorbent stability after 6 months of testing [reproduced with permission from ref (12), copyright 2019 Springer].

Recently, we established a bench-scale fluidized-bed CO_2_ capture process with a new pilot plant in collaboration with RTI International.^50,55−57^ As shown in Figure 7a, the bench-scale system contains two fluidized, moving-bed reactors (sorber and regenerator) and has the capability to treat flue gas at flow rates of 300–900 SLPM (standard liter per minute) with solid sorbents of 75–450 kg/h. The MBS sorbent was prepared in a large quantity (over 100 kg) in a toll manufacturer with our prescribed procedure. MBS sorbent is continuously circulated as fine particles between the two reactors. Typically, about 75 kg of sorbent was loaded, capturing CO_2_ at ca. 150 kg of CO_2_/day. During the 100 h operation, 90 ± 2% of CO_2_ was captured from the flue gas. The performance was sustained over the testing period (Figure 7b, the large fluctuation is due to operating condition change). Furthermore, after 6 months of operation, the performance of the spent sorbent had no significant degradation in comparison with that of the fresh sorbent (Figure 7c), indicating good stability and regenerability of MBS. It should also be pointed out that after 6 months of testing, the attrition of sorbent particles was negligible.

After the successful tests of MBS in the bench-scale system, the same pilot plant was taken to Norway and further tests were performed using the flue gas from a cement plant.^55,57^ The results showed that the presence of 100 ppm of SO_2_ in the flue gas can cause about a 30% loss in the CO_2_ capacity of MBS over a 100 sorption–desorption cyclic operation. This indicates that preremoval of SO_2_ from the real flue gas may be required. It is consistent with the conclusion from our earlier tests in a pilot plant (demonstration boiler) with coal-fired and natural gas-fired pilot plant flue gases.^58,59^

To address the negative impact of SO_2_ and NO_2_ in real flue gas, we developed a new MBS by loading PEG onto SBA-15 to selectively capture SO_2_ and NO_2_.^26^ The sorbent can effectively remove SO_2_ and NO_2_ from 500 ppm to less than 2 ppm and is fully regenerable. Later, we studied a series of EG (ethylene glycol) derivatives and DMSO (dimethyl sulfoxide)-based MBS for SO_2_ removal.^37^ Compared with EG derivatives, the DMSO-based MBS exhibits a much higher SO_2_ sorption capacity. Additionally, it selectively removes SO_2_ from CO_2_-rich gas streams with a relative selectivity factor of 120. The high SO_2_ selectivity over that of CO_2_ can be attributed to the higher polarity of SO_2_ as DMSO is a polar solvent.

Based on the results from the bench-scale operation, a technoeconomic analysis of the CO_2_-capture process based on MBS has been conducted. The estimated CO_2_ capture cost was around $43.3/ton of CO_2_,^55^ much lower than that of the amine scrubbing process ($70 ± $15/ton of CO_2_^7,60^). Although it has not reached the goal of the U.S. DOE’s Carbon Capture Program, which is < $40/ton of CO_2_ by 2025, the bench-scale study has shown the promise of MBSs and the possibility of improving MBSs, with the aim being the U.S. DOE’s cost target.

## Summary and Outlook

Developing new and more efficient CO_2_ capture technology is both a difficult challenge and an urgent task for the CCUS and sustainable development. To overcome the drawbacks of the conventional liquid amine scrubbing process, using a new type of effective solid (ad)sorbents holds great promise. This Account showcases our development of the molecular basket sorbent (MBS), a polymeric amine embedded in solid porous materials, for CO_2_ capture. MBS is promising for real applications as it has many advantages including high CO_2_ capacity even at extremely low CO_2_ concentrations, excellent CO_2_ selectivity, a positive moisture effect, lower energy consumption, less corrosion and easy handling compared to liquid amine solution, good regenerability, and stability.

We highlight the significance of fundamental research in the development of next-generation carbon capture technologies with improved energy efficiency and cost-effectiveness. The application of modern in situ characterization techniques such as STEM, SANS, and CO_2_-TPD enabled us to characterize the spatial distribution and deposition morphology of PEI and identify the key obstacle within MBSs for CO_2_ capture, i.e., the aggregated PEI plugging the pore channels. It is also the reason for its unique temperature dependence on the CO_2_ capacity and sorption kinetics. Consequently, we were able to develop new strategies to overcome the barrier and greatly improve the sorbent performance. That includes altering the temperatures for sorption and desorption; using support materials with larger surface area, large pore volume, and unique pore structure (e.g., 3-D pores, inverted cone-shaped pores); specifically arranging the amine assembly within the pore channels; altering the type of amine polymer; and incorporating additives such as PEG, APTES, and K_2_CO_3_.

One great milestone in advancing MBSs is that the MBSs have been tested for CO_2_ capture at pilot scales with real flue gases from coal-fired and natural gas-fired boilers and from a cement plant. The test results are promising and show the potential of MBS for commercial deployment. The estimated overall cost for the capture of CO_2_ from flue gas using MBS was about $43/ton-CO_2_. The studies also showed, however, that SO_*x*_ and NO_*x*_ can also be adsorbed on MBS for CO_2_, which could degrade the sorbent during long-term operation. This means that preremoval of SO_*x*_ and NO_*x*_ may be needed for MBS. That is the reason that we have also developed specific MBS that can selectively capture SO_2_ and NO_2_ from flue gas streams in the presence of CO_2_ and N_2_.^29^

Through the past two decades of research, we have made some significant progress, but more research and development are needed to further reduce the cost and make the process more competitive; the sorption kinetics and cyclic sorption capacity along with the stability need further improvement. How to design the system with the consideration of sorption kinetics and regeneration cycles is another aspect to consider. We consider the following challenges and research needs:

1. Reducing the sorbent cost is crucial to decreasing the overall CO_2_ capture cost. Currently, most research and development focus on novel support materials, but it may require time and an expensive procedure for the synthesis. Finding inexpensive materials with a simple preparation procedure would be more desirable and would require more attention in research and development.
2. Although several models have been proposed, CO_2_ sorption and desorption kinetics on supported PEI sorbents are not well addressed, including sorption and desorption rates under various conditions, particularly under real conditions in the presence of moisture, SO_2_, NO_*x*_, and O_2_. This is closely related to the CO_2_ capture process design and the choice of reactor configurations (i.e., fluidized bed, moving bed, packed bed, etc.). The adsorption of moisture, SO_2_, and NO_*x*_ and amine oxidation because of O_2_ could induce the change in PEI distribution and morphology, which in turn influences the CO_2_ sorption capacity and sorption/desorption rates.
3. We observed the promotion effect of moisture on the CO_2_ sorption over MBSs, but the effect of moisture on CO_2_ desorption has had little study. How the presence of moisture affects the spatial distribution and morphology of PEI, the CO_2_ sorption enthalpy and rate, the energy needed for sorbent regeneration after moisture sorption, and sorbent long-term stability is seldom reported. It is an important topic, especially when steam-stripping is considered to be an option for regenerating sorbent and recovering CO_2_ for utilization and storage.
4. Many studies focus on CO_2_ capture from large point sources while some studies focus on capture from air. Distributed and dispersed CO_2_ emissions such as in the transportation sector can contribute up to one-third of total CO_2_ global emissions, but little attention is being paid to CO_2_ capture from transportation on land, sea, and air. Although direct air capture is attracting increasing attention, it is challenging due to extremely low CO_2_ concentration in the air (i.e., ∼410 ppm). Designing a mobile CO_2_ capture processor with MBSs could be a promising alternative and a complementary solution but requires more research in this area.

Finally, it should be noted that although this Account focuses on the molecular basket sorbents, the principles discussed should be applicable to most other supported amine sorbents for CO_2_ capture. We hope this Account will help pave the way and shed light on the future development of new CO_2_ capture technologies based on adsorption.
